# BluB/CobT2 fusion enzyme activity reveals mechanisms responsible for production of active form of vitamin B_12_ by *Propionibacterium freudenreichii*

**DOI:** 10.1186/s12934-015-0363-9

**Published:** 2015-11-23

**Authors:** Paulina Deptula, Petri Kylli, Bhawani Chamlagain, Liisa Holm, Risto Kostiainen, Vieno Piironen, Kirsi Savijoki, Pekka Varmanen

**Affiliations:** Department of Food and Environmental Sciences, University of Helsinki, 00014 Helsinki, Finland; Division of Pharmaceutical Chemistry and Technology, University of Helsinki, 00014 Helsinki, Finland; Institute of Biotechnology, University of Helsinki, 00014 Helsinki, Finland

**Keywords:** *Propionibacterium freudenreichii*, Cobalamin, B12, DMBI, α-Ribazole, Phosphoribozyltransferase, Nitroreductase, Fusion enzyme, BluB, CobT

## Abstract

**Background:**

*Propionibacterium freudenreichii* is a food grade bacterium that has gained attention as a producer of appreciable amounts of cobalamin, a cobamide with activity of vitamin B_12_. Production of active form of vitamin is a prerequisite for attempts to naturally fortify foods with B_12_ by microbial fermentation. Active vitamin B_12_ is distinguished from the pseudovitamin by the presence of 5,6-dimethylbenzimidazole (DMBI) as the lower ligand. Genomic data indicate that *P. freudenreichii* possesses a fusion gene, *bluB*/*cobT2*, coding for a predicted phosphoribosyltransferase/nitroreductase, which is presumably involved in production of vitamin B_12_. Understanding the mechanisms affecting the synthesis of different vitamin forms is useful for rational strain selection and essential for engineering of strains with improved B_12_ production properties.

**Results:**

Here, we investigated the activity of heterologously expressed and purified fusion enzyme BluB/CobT2. Our results show that BluB/CoBT2 is responsible for the biosynthesis of the DMBI base and its activation into α-ribazole phosphate, preparing it for attachment as the lower ligand of cobalamin. The fusion enzyme was found to be efficient in metabolite channeling and the enzymes’ inability to react with adenine, a lower ligand present in the pseudovitamin, revealed a mechanism favoring the production of the active form of the vitamin. *P. freudenreichii* did not produce cobalamin under strictly anaerobic conditions, confirming the requirement of oxygen for DMBI synthesis. In vivo experiments also revealed a clear preference for incorporating DMBI over adenine into cobamide under both microaerobic and anaerobic conditions.

**Conclusions:**

The herein described BluB/CobT2 is responsible for the production and activation of DMBI. Fusing those two activities results in high pressure towards production of the true vitamin B_12_ by efficiently activating DMBI formed within the same enzymatic complex. This indicates that BluB/CobT2 is the crucial enzyme in the B_12_ biosynthetic pathway of *P. freudenreichii*. The GRAS organism status and the preference for synthesizing active vitamin form make *P. freudenreichii* a unique candidate for the in situ production of vitamin B_12_ within food products.

**Electronic supplementary material:**

The online version of this article (doi:10.1186/s12934-015-0363-9) contains supplementary material, which is available to authorized users.

## Background

**Fig. 1:**
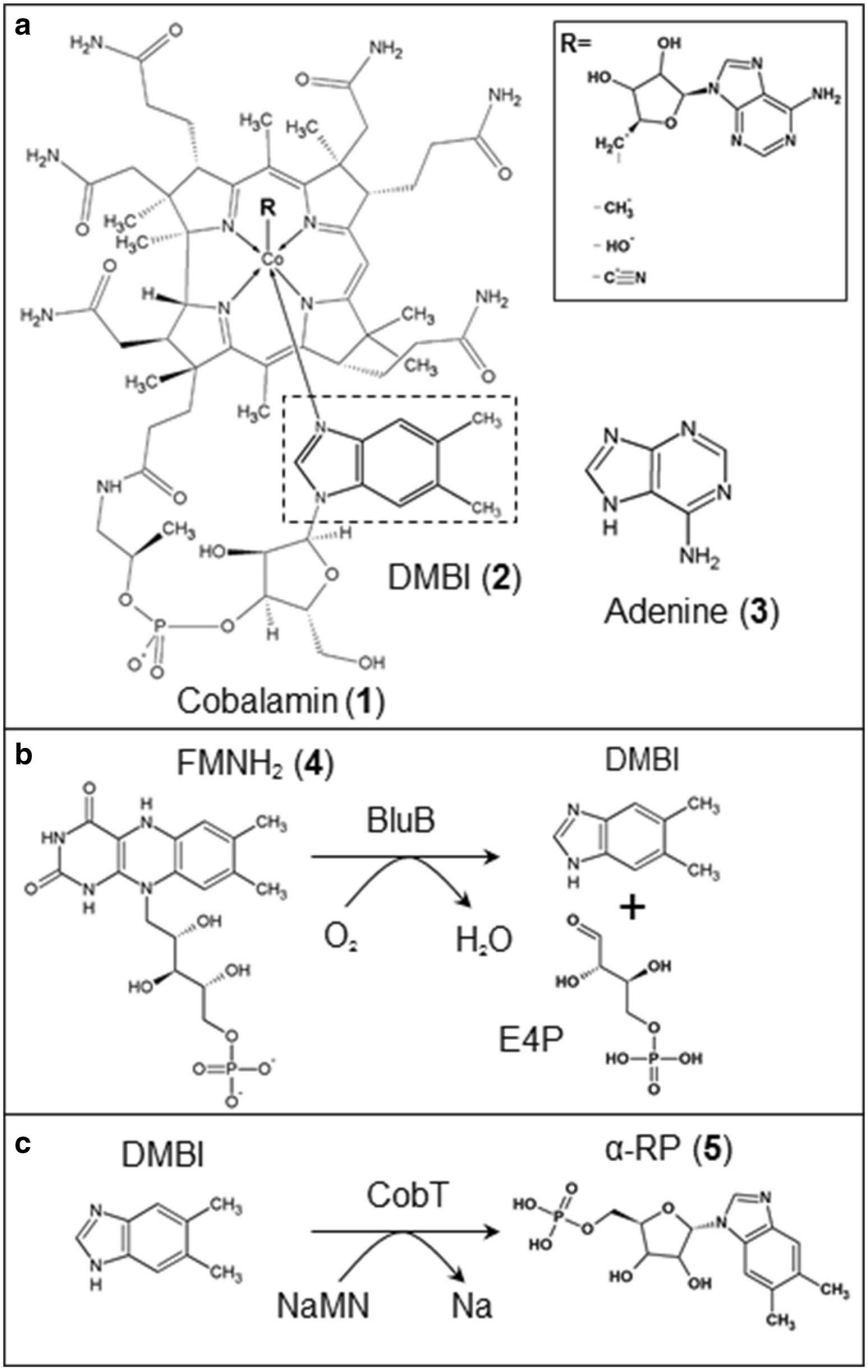
Chemical structures. **a** Structure of cobalamin (**1**) molecule with the lower ligand DMBI (**2**) indicated by a dotted box. Adenine (**3**) is the lower ligand of the pseudovitamin form. R denotes possible upper ligands. **b** The reaction catalyzed by the BluB enzyme: formation of DMBI from FMNH_2_ (**4**). **c** The reaction catalyzed by the CobT enzyme: formation of α-RP (**5**) from DMBI and NaMN

Cobamides are corrinoid compounds and essential cofactors synthesized uniquely by certain microorganisms. Their characteristic structure consists of a corrin ring with a central atom of cobalt and two axial ligands coordinated to the cobalt, the so-called upper and lower ligands. Among cobamides, cobalamins, distinguished by the presence of 5,6-dimethylbenzimidazole (DMBI) as a lower ligand (Fig. 1a), are of special human nutritional value because of their activity of vitamin B_12_. Vitamin B_12_ plays an important role in nucleotide synthesis as well as the metabolism of branched amino acids and odd-chain fatty acids. These molecules fulfill their role as cofactors in cytoplasmic methionine synthase and in mitochondrial methylmalonyl-CoA mutase, which are responsible for the methylation of homocysteine to methionine and the conversion of methylmalonyl-CoA to succinyl-CoA, respectively [1]. Cobalamin deficiency may lead to megaloblastic anemia and/or neuropsychiatric complications and, left untreated, can result in irreversible neurological disorder or even death [2]. DMBI as a lower ligand is crucial for the binding of cobalamin to the intrinsic factor, a glycoprotein responsible for its transport in the human gastrointestinal tract [1]. Due to the complexity and cost of the chemical synthesis of cobalamin, which requires approximately 70 steps, its industrial production is exclusively through microbial fermentation, most commonly by *Pseudomonas denitrificans* and *Propionibacterium freudenreichii* [3].

In recent years the interest in fortification of food products in vitamin B12 by in situ fermentation has been growing [4–8]. Vitamin B_12_ production by *P. freudenreichii* is of special interest because of the Generally Recognized as Safe (GRAS) status of the organism, which allows its direct use in food and feed preparations [3]. Additionally, *P. freudenreichii* is an efficient producer, reaching cobalamin levels of 15 µg/mL [9], and furthermore, reportedly produces only trace amounts of pseudocobalamin [10]. In the pseudo form of the vitamin the lower ligand, adenine (Fig. 1a), renders its affinity to intrinsic factor 500-fold lower than cobalamin [11]. Some other GRAS organisms, mainly *Lactobacillus*, have also been reported to produce cobalamin [12–15]; however, it is questioned whether these species produce the active or the pseudo form of vitamin. *Lactobacillus reuteri,* which possesses the genes for B_12_ synthesis [13], was recently shown to produce pseudocobalamin exclusively due to its inability to incorporate other bases than adenine as the lower ligand of the cobamide [16, 17]. *Streptomyces griseus* produces vitamin B_12_ for which the GRAS status has been granted (§184.1945), however since “Streptomyces spp. produce antibiotics and are therefore inappropriate for QPS (EFSA opinion 2008)” [18] the bacterium itself is not eligible for such status. With Lactobacilli unable to produce active vitamin B_12_, *P. freudenreichii* remains the only known producer with GRAS status granted by the FDA, and QPS granted by EFSA [18] allowing for its direct use in food and feed preparations.

The complete biosynthesis of cobalamin requires approximately 30 gene products [3], but it is the final steps of the pathway, namely production, activation and attachment of the lower ligand that decide whether the final product will be an active vitamin B_12_ or an analog. In aerobic and aerotolerant microorganisms, DMBI is synthesized in an oxygen-dependent manner from reduced FMN by the action of the BluB enzyme [19–21], while in strict anaerobes gene cluster *bzaABCDE* has been recently associated with this function [22].

Synthesized DMBI is then activated by the CobT enzyme (CobU in aerobes) into α-ribazole-phosphate (α-RP) (Fig. 1c), preparing the lower ligand for attachment to form the complete cobalamin molecule [23]. Recent studies determined that the selectivity of CobT is responsible for the range of lower ligands that can be attached to cobamide [17]. A study with seven CobT homologues from diverse organisms revealed that DMBI is a preferred substrate (with the exception of ArsAB of *Veillonella parvula* preferentially activating phenolic bases), although some of the homologues, such as CobT from *L. reuteri,* were unable to activate DMBI in vivo [24]. In a related report, it was suggested that the ability to exclude certain lower ligands from incorporation into cobamides provides a mechanism preventing the production of compounds that cannot be used by the organism [17].

The genetics of the complete cobalamin synthesis pathway of *P. freudenreichii* have been previously studied and described [25–30]. The genome sequence of *P. freudenreichii* CIRM-BIA^1^ indicated that in this organism, the *bluB* gene is fused with *cobT2* [30] resulting in a putative phosphoribosyl- transferase/nitroreductase that could both synthesize and activate DMBI for attachment into the cobalamin molecule. Although homologues of the fusion enzyme BluB/CobT2 have been found in multiple organisms on the genetic level, the enzyme has not yet been studied. In this work, we used the heterologously expressed and purified BluB/CobT2 enzyme of the type strain *P. freudenreichii* subsp. *shermanii* DSM 4902 to study its predicted novel ability to both synthesize and activate DMBI for attachment as a lower ligand in the final steps of biosynthesis of the active vitamin B_12_.

## Results and discussion

### Heterologous expression and purification of the BluB/CobT2 enzyme

**Fig. 2:**
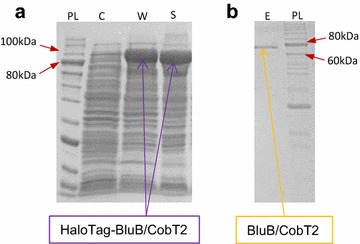
SDS-PAGE analysis of the purified recombinant BluB/CobT2 enzyme. **a** Control of expression and solubility of the HaloTag-BluB/CobT2: uninduced cells (*lane C*), whole-cell lysates (*lane W*), soluble fraction of the lysates (*lane S*). **b** Purified, tag-free BluB/CobT2 (*lane E*). Protein marker (*lanes* marked *PL*), stained with PageBlue

The *bluB/cobT2* coding region [NCBI: NC_014215.1] was cloned in the pFN18A vector and transformed into *Escherichia coli* KRX competent cells. From selected *E. coli* clones the inserts were sequenced to exclude alterations in the sequence. The rhamnose-induced heterologous expression and solubility of the recombinant protein were confirmed by SDS-PAGE (Fig. 2a). The predicted size for HaloTag-BluB/CobT2 protein is 100.4 kDa (34 kDa HaloTag + 66.4 kDa BluB/CobT2) and a band of corresponding molecular weight was detected in samples from *E. coli* whole-cell lysates and soluble fraction of the lysates obtained after induction (Fig. 2a). Following purification steps and tag removal the purified BluB/CobT2 was obtained (Fig. 2b). The resulting BluB/CobT2 recombinant protein contains only four extra amino acids (SDNA) in its N-terminus, replacing the initial methionine of native BluB/CobT2 of *P. freudenreichii*. The concentration of the obtained protein preparate was 0.4 mg/ml.

### Sequence analysis on BluB/CobT2

**Fig. 3:**
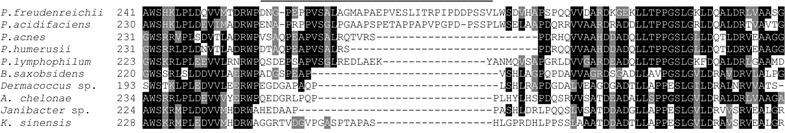
Alignment of the spacer regions. Part of the multiple sequence alignment of predicted BluB/CobT fusion proteins from different microorganisms showing the region between the BluB (aa 1–257) and CobT (aa 294–626) domains of BluB/CoBT2 of *P. freudenreichii.* The predicted spacer region is indicated by a *line* above the amino acid sequence. Protein sequences were obtained from NCBI Protein data base: *P. freudenreichii* [WP_013160556.1], *P. acidifaciens* [WP_028706153.1], *P. acnes* [WP_002548940.1], *Propionibacterium humerusii* [WP_007433757.1], *P. lymphophilium* [EPD33903], *Blastococcus saxobsidens* [WP_014375557.1], *Dermacoccus* sp. [WP_047312757.1], *A. chelonae* [WP_006501272.1], *Janibacter* sp. [WP_009778314.1] and *Knoellia sinensis* [WP_035916832.1] sequences were aligned by ClustalW and rendered using BOXSHADE

The gene product BluB/CobT2 [NCBI: CBL56167.1] from *P. freudenreichii* is a 626 amino acid protein, with the N-terminal part (aa 1–257) showing 40 and 35 % identity to previously characterized BluB enzymes from *Sinorhizobium meliloti* [19] and *Bacillus megaterium* DSM319 [21], respectively. The C-terminal part (aa 294–626) of BluB/CobT2 shows ~30 % identity to previously characterized CobT enzymes from *Salmonella enterica, L. reuteri, and S. meliloti*, to ArsA from *V. parvula* [17] and to CobU from *P. denitrificans* [31]. The BluB/CobT2 regions homologous to BluB and CobT are interspaced by a proline-rich (23 %) stretch of ~34 amino acids possibly forming a spacer region between the BluB and CobT2 domains. The proline-rich sequence, typical of non-helical protein linkers, can form extended rigid structures separating the protein domains [32], but “elbow bending” dynamics can also be observed [33]. Therefore, the linker could play a role in the dimeric interaction between the protein subunits, as domains BluB and CobT2 both have predicted dimeric interfaces [34]. Alternatively, the spacer region may be a remnant of the fusion event with no specific function. The ClustalW alignment of predicted BluB/CobT fusion proteins revealed that in organisms other than *P. freudenreichii* and *Propionibacterium acidifaciens,* the region separating the BluB and CobT domains, corresponding to aa 258–293 in BluB/CoBT2, is shorter (Fig. 3).

According to a Blastp search, the closest homolog of BluB/CobT2 is *P. acidifaciens,* which encodes a 72 % identical protein [NCBI: WP_028706153.1]. Notably, BluB/CobT2 fusion proteins are not found in all members of propionibacteria. Outside propionibacteria, a Blastp search revealed the highest homology against proteins from *Propionimicrobium lymphophilum* ACS-093-V-SCH5 and *Austwickia chelonae,* showing 59 and 58 % identity with BluB/CobT2 of *P. freudenreichii*, respectively. Another Actinobacterium and known producer of vitamin B_12_, *Streptomyces griseus*, also posseses a fusion BluB/CobT enzyme, however it is additionally fused with a protein of unknown function at the N-terminus. *S. griseus* fusion protein shows 34 % identity over the whole region of homology to *P. freudenreichii* BluB/CobT2. BluB/CobT appears to be rather widespread among Actinobacteria, but only a few isolated entries from other classes, including Spirochaetia, Flavobacteria, Cytophagia and Deltaproteobacteria, were found.

### BluB/CobT2 produces DMBI in presence of FMN and NADH

For enzymatic characterization, BluB/CobT2 of *P. freudenreichii* was heterologously produced in *E. coli* KRX and purified. The obtained recombinant BluB/CobT2 was used in enzyme assays. Reactions performed with BluB/CobT2 included the formation of DMBI and its subsequent activation into α-ribazole-phosphate. We addressed these two predicted activities of BluB/CobT2 both separately and jointly.

**Fig. 4:**
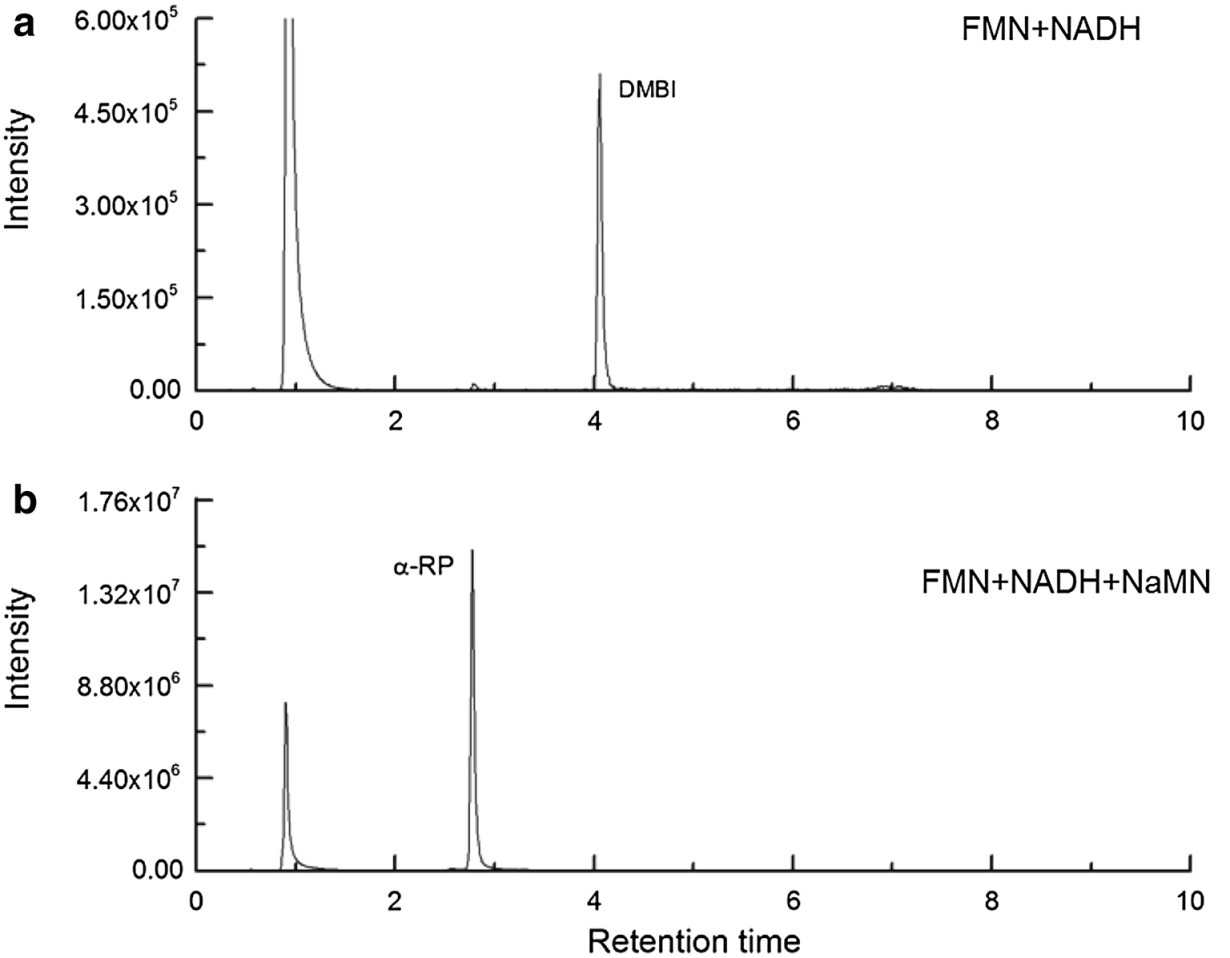
Production and phosphoribosylation (activation) of DMBI by BluB/CobT2. **a** The LC chromatogram of the DMBI synthesis reaction containing BluB/CobT2, FMN and NADH after incubation for 48 h. **b** The LC chromatogram of two-step DMBI synthesis and activation reaction producing α-RP in the presence of the BluB/CobT2, FMN, NADH and NaMN after incubation for 48 h. The peaks were verified by MS/MS analysis. See also Additional file 1: Figure S1

BluB is responsible for the formation of DMBI from FMNH_2_ in the presence of oxygen [19]. In order to reduce FMN to FMNH_2_, a flavin reductase, such as SsuE [19] and chemical reduction with NADH [19, 21] have been used previously. Here, DMBI formation by BluB/CobT2 was studied in the presence of 100 µM FMN and 0, 20 or 40 mM of NADH. Preliminary experiments indicated that pH 8.5 was the most favorable for the reaction (data not shown). Liquid chromatography electrospray ionization mass spectrometry (LC–ESI–MS) was used to identify the reaction products. A compound with the expected *m/z* ratio for DMBI (*m/z* 147.3) was observed at the retention time of 4.05 min (Additional file 1: Figure S1). A commercial standard of DMBI, which appeared at the same m/z value and retention time, was used for the identification (Fig. 4a). Only trace amounts of DMBI were detected in the reaction without NADH, and the amount of DMBI increased with increasing NADH concentration (Table 1), indicating that the reduction of FMN by NADH was one of the limiting factors of DMBI production under the conditions used. *S. enterica* CobT has also been reported to catalyze the reaction of DMBI with NAD+ [35]. However, under the conditions used, we observed no peak corresponding to DMB-adenine dinucleotide (α-DAD) as a product of this reaction.

**Table 1:** Effect of NADH concentration and presence of NaMN on levels of DMBI and α-RP in reactions with the BluB/CobT2

NADH (mM)	FMN (100 µM)	FMN (100 µM) + NaMN (200 µM)	
	DMBI (nM)	α-RP (nM)	DMBI (nM)
0	120 ± 6	–	–
20	1362 ± 12	4458 ± 200	0.5 ± 0.2
40	2301 ± 9	5095 ± 215	2 ± 0.5

The concentrations of DMBI and α-RP were measured following incubation of reactions for 48 h

The values reported are an average of three independent reactions, with standard deviation

### BluB/CobT2 activates DMBI into α-RP in the presence of NaMN

CobT catalyzes the activation of the lower ligand base to form an α-ribosylated product. For the BluB/CobT2 fusion enzyme, the lower ligand base DMBI is provided directly by the BluB part to feed the activation into α-RP by the CobT2 part of the enzyme. After confirming that BluB/CobT2 produces DMBI from FMNH_2_, we prepared a two-step reaction containing the substrates necessary for the biosynthesis of DMBI and its subsequent activation into α-RP, namely 100 µM FMN, 20 or 40 mM NADH and 200 µM of nicotinic acid mononucleotide (NaMN). LC–MS/MS analysis of the reaction products revealed a peak with the retention time of 2.75 min at *m/z* 359.1 that corresponds to α-RP (Fig. 4b). An MS/MS experiment on the ion at *m/z* 359.1 showed a fragment of 147.3, which corresponds to DMBI (Additional file 1: Figure S1). DMBI was observed only in trace amounts, and this result was not affected by NADH concentration, suggesting efficient activation of the produced DMBI into α-RP by BluB/CobT2 (Table 1). It has been noted previously that benzimidazoles do not appear to be used for any other purposes than the lower ligands of cobamides [17]. The fusion of BluB with CobT allows the efficient use of produced DMBI, which is not required for any other processes in the cell.

In general, the fusion of two activities in a single polypeptide may be beneficial for the enzyme activity because of the increased efficiency of substrate transfer, protection of the intermediates, facilitation of the interactions between domains and establishment of a fixed stoichiometric ratio of the enzymatic activities of sequential reactions [36]. In our experiments the molar concentrations of α-RP obtained from reaction coupling synthesis and activation of DMBI were ~2–3-fold higher than the DMBI concentrations measured from the DMBI synthesis reaction alone (Table 1). This may indicate that the presence of NaMN stimulates the production of DMBI, possibly through imposing certain conformational changes on the BluB/CobT2 enzyme, resulting either in more efficient release of DMBI from the active site of the BluB part or an increase in BluB enzyme activity. The increased formation of α-ribazole-phosphate in the presence of NaMN relative to the formation of DMBI can be attributed to either of these mechanisms, leading to efficient usage of the scarce substrates. This issue could be addressed further through crystallography studies.

### Effect of substrate and environmental pH on DMBI activation by BluB/CobT2

**Fig. 5:**
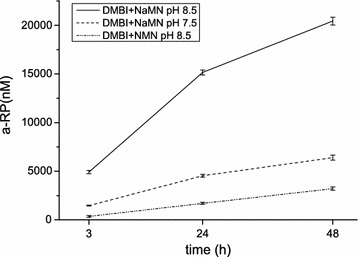
Effect of substrate and pH on α-RP production by BluB/CobT2. The *graph* showing levels of α-RP formed by the BluB/CobT2 enzyme from DMBI and NaMN at pH 7.5 and 8.5 and α-RP formed from DMBI and NMN at pH 8.5

It has been previously shown that semi-purified *P. freudenreichii* CobT requires NaMN or nicotinamide mononucleotide (NMN) in addition to DMBI for α-RP formation, with NaMN being a superior substrate producing sevenfold more α-RP than NMN [10]. In the same study, the optimal pH range for the activity of semi-purified CobT was determined to be between 8.5 and 9.4 [10]. For the CobT enzymes of *S. meliloti*, *S. enterica*, *L. reuteri*, *Desulfovibrio vulgaris*, *Dehalococcoides mccartyi* and *Veillonella parvula,* pH 7.5 has been used as a reaction condition [24]. Here, we tested α-RP formation by BluB/CobT2 from NaMN and exogenous DMBI at pH 8.5 and 7.5, resulting in more than a threefold higher concentration of α-RP formed at pH 8.5 (Fig. 5). Subsequently, we compared the preference towards NaMN and NMN at pH 8.5. The amount of α-RP formed with NaMN as a substrate was 6.4-fold higher than with NMN (Fig. 5), which is in accordance with previous results by Friedmann [10] and confirms that NaMN is the preferred substrate.

### Formation of Ade-RP by BluB/CobT2

**Fig. 6:**
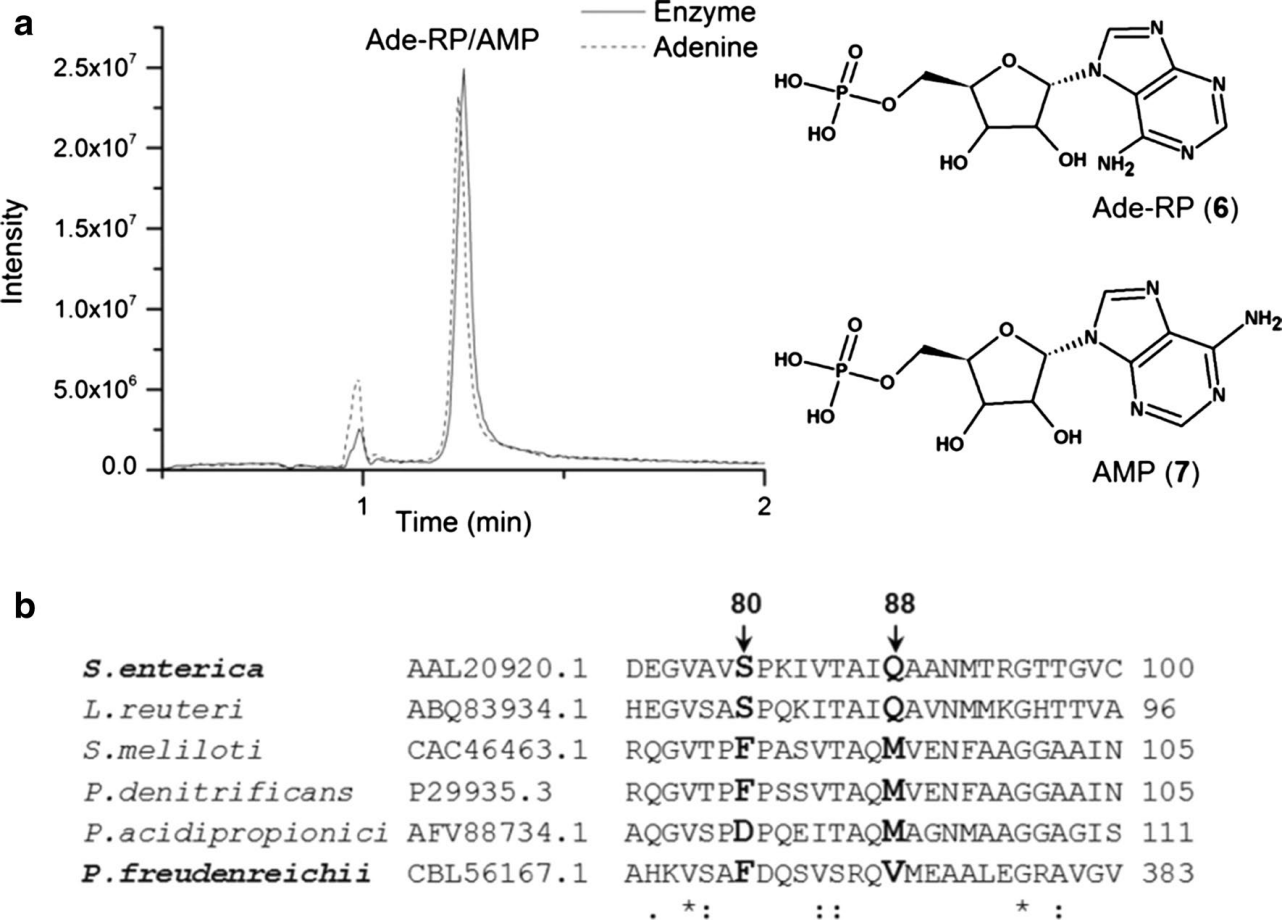
BluB/CobT2 reaction product with adenine and NaMN was not detected. **a** The LC chromatograms of the 48 h reaction with BluB/CobT, adenine and NaMN (*dotted*) at pH 8.5 and of the BluB/CobT enzyme preparation (*solid line*). Chemical structures of isomers Ade-RP and AMP indistinguishable by LC–MS/MS are shown. See also Additional file 1: Figure S2. **b** Part of the ClustalW alignment of CobT enzymes from *S. enterica* [NCBI: AAL20920.1], *L. reuteri* [NCBI: ABQ83934.1], *S. meliloti* [NCBI: CAC46463.1], *P. denitrificans* [Swiss-Prot: P29935.3], *P. acidipropionici* [NCBI: AFV88734.1] and *P. freudenreichii* [NCBI: CBL56167.1]. The amino acids corresponding to S80 and Q88 of *S. enterica* CobT and critical for lower ligand substrate specificity [17] are marked in *bold*. See also Additional file 1: Table S1

All of the CobT homologues studied thus far have had the ability to activate more than one type of base into a corresponding α-riboside phosphate, including different forms of benzimidazoles, purines and phenolic compounds, with the number and type of bases varying between enzymes from different organisms [24]. From the perspective of the biosynthesis of cobalamin, the activation of adenine is of particular interest because adenine activated into 7-α-d-ribofuranosyladenine 5′phosphate (Ade-RP) would serve as a substrate for the lower ligand of pseudocobalamin, resulting in competition between production of the active and pseudo-vitamin form. Previous studies using partially purified cell-extracts of *P. freudenreichii* have suggested that in this bacterium, the CobT-like activities do not include the activation of adenine [10, 37]. Here, the activation of adenine was tested at pH 7.5 and 8.5 in the presence of NaMN, conditions that were found suitable for the activation of DMBI. A peak with the retention time of 1.25 min at *m/z* 348 (Additional file 1: Figure S2) and with the same size was detected in reactions with and without adenine and in the sample with enzyme alone. This peak is most likely due to ATP used in the purification process of the enzyme, leading to residual AMP in the BluB/CobT2 preparation. Because 9-β-d-ribofuranosyladenine 5′phosphate (AMP) and Ade-RP are isomers (Fig. 6a), they cannot be distinguished by MS/MS. However, because the addition of adenine in the reaction did not increase the size of the Ade-RP/AMP peak, we conclude that BluB/CobT2 does not react with adenine under the conditions tested. It was previously shown that the *P. freudenreichii* cell extracts form DMBI from FMN [38] and activate DMBI rather than adenine for attachment as a lower ligand into cobamide [10] however, the mechanisms behind this preference remained obscure. One possible explanation was provided by structural studies of CobT from *S. enterica,* which revealed that two polar amino acids stabilize adenine in the active site: serine at position 80 interacts with the amino group (N10) and glutamine at position 88 interacts with the N3 ring nitrogen of adenine [17, 39]. Both of these groups are different in DMBI (Fig. 1a), and therefore mutations of S80 and Q88 show different preferences of DMBI versus adenine activation. DMBI lacks the amino group and has a CH group instead of the N3 ring nitrogen. Crofts et al. [17] showed that changing serine to phenylalanine at position 80 decreased the reaction of CobT with adenine, while changing glutamine to methionine at position 88 decreased the reaction with adenine and furthermore increased the reaction with DMBI. The effect of these amino acid changes from hydrophilic to hydrophobic residues were found to be additive, as *S. meliloti* expressing the CobT enzyme of *S. enterica* carrying both mutations was found to produce the lowest amount of pseudo-cobalamin and the highest amount of cobalamin [17]. In the present study, multiple sequence alignment of *P. freudenreichii* BluB/CobT2 with other enzymes revealed the hydrophobic residues phenylalanine and valine at positions corresponding to S80 and Q88 in *S. enterica* CobT (Fig. 6b). Similar organization is present in CobT proteins of *S. meliloti* and *P. denitrificans*, two known producers of cobalamin, which carry hydrophobic phenylalanine and methionine in these positions (see also Additional file 1), further implying the role in substrate specificity. Considering that *P. freudenreichii* has been reported to produce small amounts of pseudocobalamin [38] but no activation of adenine by *P. freudenreichii* BluB/CobT2 was observed, the reaction leading to the pseudo form may be catalyzed by another, yet unidentified enzyme [10, 38]. Several bacteria possess another enzyme capable of activating cobamide bases, such as ArsA and ArsB, which are homologous to CobT, but activate phenolic bases as well [40], while species such as *Propionibacterium acnes* and *Moorella thermoacetica* have been reported to possess two seemingly redundant CobT enzymes [41], which could have different substrate specificities. No such enzyme has been identified in *P. freudenreichii* thus far, therefore the importance of hydrophobic amino acids at positions corresponding to S80 and Q88 in *S. enterica* CobT for substrate specificity in BluB/CobT2 of *P. freudenreichii* should be addressed in targeted amino acid substitutions in future studies.

### Cobamide production in vivo

Previous studies have revealed that the range of cobamides produced by a given organism in vivo does not always correlate to the range observed with CobT enzymes in vitro. Furthermore, these studies have shown that the cobamide requirements of the organism largely determine the variety it produces [17, 24]. An attempt to distinguish between cobalamin and pseudocobalamin production under aerobic and strictly anaerobic conditions was previously investigated in *S. enterica,* showing that the bacterium exclusively synthesizes pseudocobalamin under strictly anaerobic conditions and cobalamin under microaerobic conditions [42]. This finding is consistent with the necessity of oxygen for the formation of DMBI in *S. enterica* [43], even though in that case the DMBI formation could have been non-enzymatic [44]. Knowing that *P. freudenreichii* is an aerotolerant anaerobe and that oxygen is required for the synthesis of DMBI, we decided to test cobamide formation in vivo under both anaerobic and microaerobic conditions.

**Fig. 7:**
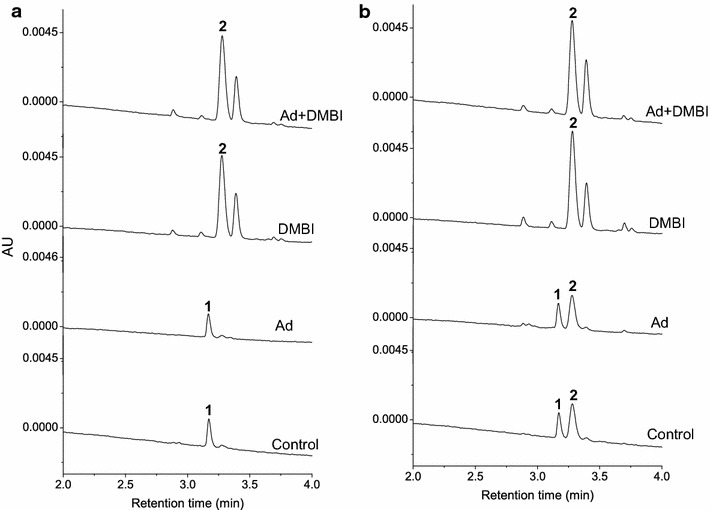
Cobamide production by *P. freudenreichii* in vivo. The UHPLC chromatograms of cobamides extracted and purified from cultures grown under anaerobic (**a**) and microaerobic (**b**) conditions, with and without addition of DMBI (100 µM) and/or adenine (Ad; 100 µM). Identity of pseudocobalamin (peak 1) and cobalamin (peak 2) were confirmed by LC–MS/MS. See also Additional file 1: Figure S3

As expected, the UHPLC (Ultra-high Performance Liquid Chromatography) analysis of cobamide extracts revealed no cobalamin but a small amount of pseudocobalamin in samples in cultures grown under strictly anaerobic conditions and without exogenous DMBI (Fig. 7; Additional file 1: Figure S3). In cultures grown under microaerobic conditions without exogenous DMBI, both cobalamin and pseudocobalamin were detected. In all culture conditions where exogenous DMBI (100 µM) was supplied, only cobalamin and no pseudocobalamin was detected, suggesting that DMBI synthesis was a limiting factor in cobalamin production under the growth conditions used. However, supplementing the growth medium with adenine (100 µM) did not increase the levels of pseudocobalamin formed, even under anaerobic conditions; instead, cobinamide, a cobamide missing the lower ligand, was detected (Additional file 1: Figure S4), implying both that *P. freudenreichii* preferentially produces the active form of vitamin B_12_ and also that the production of DMBI is the limiting factor in this process.

Finally, we observed no peak corresponding to pseudocobalamin in the samples from cells grown under conditions where both bases were supplemented, indicating a preference towards incorporating DMBI over adenine and thereby a preference for the production of the active vitamin over the pseudovitamin in vivo (Fig. 7). For *S. meliloti,* pseudocobalamin is not functionally equivalent to cobalamin, as observed from the poor growth of mutant strains producing only the pseudo form of the vitamin in guided biosynthesis [17]. For *P. freudenreichii,* we observed that the cultures reached the same final cell densities under anaerobic conditions with only the pseudovitamin produced, suggesting that while pseudocobalamin is not the preferred corrinoid it may, under some conditions, be able to substitute for cobalamin.

## Conclusions

The herein described fusion enzyme BluB/CobT2 is responsible for the production and activation of DMBI for attachment as a lower ligand for cobalamin. Fusing those two activities results in high pressure towards the production of true vitamin B_12_ by activating DMBI produced within the same enzymatic complex. Presence of NaMN was shown to stimulate the production of DMBI in vitro, suggesting a regulatory mechanism securing efficient usage of the scarce substrates. This efficiency, combined with the low ability to produce the pseudovitamin B_12_ form in vivo, suggests that *P. freudenreichii* may prefer the active vitamin form as a cofactor for its own use. Our results indicate that BluB/CobT2 is the crucial enzyme in the B_12_ biosynthetic pathway, responsible for the production of active vitamin over pseudovitamin. At present, *P. freudenreichii* is the only microorganism that has been both granted GRAS status and shown to produce the active form of vitamin B_12_, making it a unique candidate for the in situ microbial fortification of food products.

## Methods

### Strains and culture conditions

The *P. freudenreichii* DSM 4902 was grown on PPA medium composed of 5.0 g tryptone (Sigma-Aldrich), 10.0 g yeast extract (DIFCO Becton, Dickinson), 14.0 ml 60 % w/w DL-sodium lactate (Sigma-Aldrich) per litre with/without 1.5 % agar, pH 7.3. For liquid cultures, the pH of the PPA medium was adjusted to 6.7 prior to autoclaving. The strain was routinely grown at 30 °C in anaerobic jar on plates and under microaerobic condition in broth, unless stated otherwise.

*Escherichia coli* KRX clones carrying pFN18A-bluB/cobT2 constructs were routinely grown in LB medium [45] with 100 µg/ml ampicillin, at 37 or 25 °C, with 275 rpm shaking.

### Overexpression and purification of BluB/CobT2

BluB/CobT2 was expressed in *E. coli* KRX cells using the Flexi^®^ Vector pFN18A (Promega, Wisconsin, USA). Genomic DNA from *P. freudenreichii* was isolated as described previously [46]. The *bluB/cobT2* coding region (NC_014215.1) was PCR amplified using primer 1 and primer 2 with SgfI and PmeI restriction sites (Additional file 1: Table S2), and cloned as a SgfI-PmeI fragment in the pFN18A vector (Promega, Wisconsin, USA). *E. coli* KRX clones carrying pFN18A-bluB/cobT2 constructs were screened by PCR using primer 3 and primer 4. Plasmid from a selected clone was further verified by sequencing of the insert at the DNA sequencing laboratory (Institute of Biotechnology, University of Helsinki).

The heterologous expression and purification of the BluB/CobT2 enzyme were performed according to the HaloTag protein expression and purification system manuals (Promega), with 50 mM NaCl, 1 mM DTT, and 0.5 mM EDTA in the purification buffer and 2 mM ATP, 10 mM MgCl and 0.005 % IGEPAL CA-630 added for the binding step at 4 °C for 16 h. The HaloTag protein was removed with HaloTEV protease (Promega, Wisconsin, USA) by incubating the protein preparation for 12 h at 4 °C, followed by 90 min incubation at room temperature. The purity of the recombinant BluB/CobT2 protein was confirmed by SDS-PAGE, stained with PageBlue (Fermentas, Thermo Fisher Scientific, Delaware, USA) and the concentration determined using a NanoDrop 1000 (Thermo Fisher Scientific, Delaware, USA. The protein was stored in aliquots at −20 °C until used.

### Sequence analyses

Similarity searches for BluB/CobT2 were performed using BLAST at the National Center for Biotechnology Information. Protein sequence alignments were performed with the program ClustalW via the ClustalW web service at the European Bioinformatics Institute [47].

### BluB/CobT enzyme activity reactions and the LC–MS method

All the enzyme activity reactions were conducted in triplicate, using 5 µM of the BluB/CobT2 enzyme. The BluB reaction was performed with 1 mM DTT, 100 µM FMN, and 20 or 40 mM NADH in 90 mM Tris–HCl at pH 8.5. CobT activity with DMBI was tested with 1 mM DTT, 100 µM DMBI, and 200 µM NaMN or NMN in 90 mM Tris–HCl at pH 7.5 or 8.5. For the measurement of CobT activity with adenine, DMBI was replaced by adenine (100 µM) in the reaction, and NMN was omitted. The two-step BluB-CobT reaction was performed with 1 mM DTT, 100 µM FMN, 20 or 40 mM NADH, and 200 µM NaMN in 90 mM Tris–HCl at pH 7.5 or 8.5. Control reactions lacking the enzyme were also performed, and in the case of BluB activity, a reaction condition lacking NADH was also tested. For the reactions containing FMN, dark tubes were used. All the reactions were incubated at room temperature, protected from direct light. After the indicated incubation time, the reactions were stopped by the addition of 6.5 % TCA.

The LC–ESI–MS consisted of a Waters Acquity UPLC I class binary solvent manager, sample manager and column thermostat (maintained at 25 °C) and a Waters Xevo TQ-S triple quadrupole mass spectrometer (Waters, Milford, MA, USA). A Waters Acquity UPLC BEH C18 (2.1 × 50 mm, 1.7 μm) column was used at a flow rate of 0.3 mL/min. The mobile phase consisted of 0.1 % formic acid in water (A) and 0.1 % formic acid in methanol (B). The linear gradient elution for DMBI and α-RP was as follows: 0–6.0 min: 5 % B to 50 % B, 6.0–6.5 min: 50 % B to 100 % B, 6.5–8.0 min: 100 % B, 8.0–8.1 min: 100–5 % B, 8.1–10.0 min: 5 % B. The linear gradient elution for adenine and adenosine monophosphate was as follows: 0–1.7 min: 0.5 % B to 1 % B, 1.7–2.2 min: 1 % B to 5 % B, 2.2–2.4 min: 5 % B to 100 % B, 2.4–3.5 min: 100 % B, 3.5–3.6 min: 100–0.5 % B, 3.6–5.0 min: 0.5 % B. The injection volume was 1 μL. The mass spectrometer was operated in the positive electrospray ionization mode: capillary voltage was 0.5 kV, source offset 50 V and cone voltage 30 V. The source temperature was 150 °C, the desolvation temperature 350 °C and the desolvation gas flow was 800 L/h. The LC–MS analyses were conducted using selected reaction monitoring (SRM): ion transitions and collision energies were as follows: DMBI 147 → 132, 25 eV; α-RP 359 → 147, 20 eV; adenine 136 → 119, 20 eV; and adenosine monophosphate 348 → 136, 25 eV.

Stock solutions (5 mM) were prepared by dissolving the analytes in deionized water. Working standard solutions were prepared by diluting the stock solutions to the appropriate concentrations. The standard solutions were used to prepare a calibration curve with the following concentration levels: 0.1, 0.2, 1.0, 2.5, 5.0, 10, 50, 100, 500, 1000, 5000 and 7500 nM. DMBI and adenine were quantitated against authentic standard compounds. Due to the lack of standard compounds, α-RP and ade-RP were quantified using DMBI and adenine as reference standards.

### Cobamide production in vivo and the UHPLC-UV/MS method

Fresh colonies of *P. freudenreichii* DSM 4902 were inoculated into 30 mL of PPA broth supplemented with 5 µg/mL of cobalt chloride and either DMBI (100 µM) or adenine (100 µM), or a combination of both DMBI and adenine. Additionally, control cultures with neither adenine nor DMBI were simultaneously inoculated. The cultures were incubated statically under normal atmosphere (microaerobic condition) or in an anaerobic chamber (Don Whitley Scientific, West Yorkshire, UK) for 7 days. For each condition, three biological replicate cultures were used. Cells were harvested by centrifugation, and the cobamides were analyzed as previously described [6]. Briefly, the cobamides were extracted, in their cyano form, from bacterial pellets by boiling with 10 mL of extraction buffer (pH 4.5) containing 100 µL of 1 % NaCN. After purification through immunoaffinity columns (Easy Extract; R-Biopharma, Glasgow, Scotland), the extracts were analyzed by UHPLC on a Waters UPLC system (Waters, Milford, MA, USA) with separation on a Waters Acquity HSS T3 C18 column (2.1 × 100 mm, 1.8 µm) at a flow rate of 0.32 mL/min and with UV detection by a photo diode array (PDA) detector at 361 nm. The mobile phase was a gradient flow of 0.025 % trifluoroacetic acid in water and 0.025 % trifluoroacetic acid in acetonitrile [6]. The injection volume was 10 μL. Cobamides were identified with their retention times and their absorption spectra, and quantified using a calibration curve prepared with a set of cyanocobalamin standards with concentrations ranging from 0.015‒1.5 ng/µL. The flowthrough from the immunoaffinity purification was also analyzed with UHPLC and MS for the presence of cobamides and cobinamide (See Additional file 1: Figure S4).

To confirm the identity of the cobamides and cobinamide, the extracts were analyzed using a high resolution quadrupole time-of-flight mass spectrometer (QTOF, Synapt G2-Si; Waters, Milford, MA, USA) with an electrospray ionization interface to the UHPLC system. The mobile phase for MS contained 0.1 % formic acid instead of 0.025 % trifluoroacetic acid. The mass spectrometer was operated in positive electrospray ionization mode with a scanning range set for ions with *m/z* of 50–1500. Parent ions were collected and fragmented (MS/MS) using argon as a collision gas. The capillary voltage was 0.5 kV, the sampling cone voltage 40 V and the source offset 80 V. The source temperature was 150 °C, desolvation temperature 600 °C, desolvation gas flow 1000 L/h, nebulizer gas flow 6.5 bar, and cone gas flow 50 L/h. The trap collision energy ramp was 15‒90 eV, trap gas flow 2 mL/min and scan time 0.2 s. A lock-spray mass correction standard (leucine-enkephalin; *m/z* 556.2771) was introduced every 10 s.

## Additional file

10.1186/s12934-015-0363-9 This file consists of four supplemental figures and two supplemental tables. Figures S1 and S2 present additional information on reactions shown in Figs. 1 and 4, including structures of compounds, retention times and fragmentation spectra. Table S1 groups CobT homologues from various microorganisms and their activities. More detailed findings of bioinformatic analysis of amino acids in positions corresponding to S80 and Q88 of CobT from *Salmonella enterica* (SeCobT) as predictors of the activity are also provided. Figure S3 presents UHPLC-UV (361 nm) chromatogram of cobamide extract after purification on immunoaffinity column and the UHPLC-MS/MS spectra of peaks identified as cobalamin and pseudobobalamin. Figure S4 shows UHPLC-UV (361 nm) chromatograms of the flowthrough which passed through the immunoaffinity columns and the UHPLC-MS/MS spectra of cobinamides. Table S2 lists primers used for PCR amplification of the *bluB/cobT2* coding region.
