# Supplementary material for: BluB/CobT2 fusion enzyme activity reveals mechanisms responsible for production of active form of vitamin B12 by Propionibacterium freudenreichii
Source: Microb Cell Fact. 2015 Nov 23;14:186. doi: 10.1186/s12934-015-0363-9 (PMC4657239; doi:10.1186/s12934-015-0363-9)
Supplement: Supplementary file 1 — 10.1186/s12934-015-0363-9 This file consists of four supplemental figures and two supplemental tables. Figures S1 and S2 present additional information on reactions shown in Figs. 1 and 4, including structures of compounds, retention times and fragmentation spectra. Table S1 groups CobT homologues from various microorganisms and their activities. More detailed findings of bioinformatic analysis of amino acids in positions corresponding to S80 and Q88 of CobT from Salmonella enterica (SeCobT) as predictors of the activity are also provided. Figure S3 presents UHPLC-UV (361 nm) chromatogram of cobamide extract after purification on immunoaffinity column and the UHPLC-MS/MS spectra of peaks identified as cobalamin and pseudobobalamin. Figure S4 shows UHPLC-UV (361 nm) chromatograms of the flowthrough which passed through the immunoaffinity columns and the UHPLC-MS/MS spectra of cobinamides. Table S2 lists primers used for PCR amplification of the bluB/cobT2 coding region. [file 12934_2015_363_MOESM1_ESM.pdf]

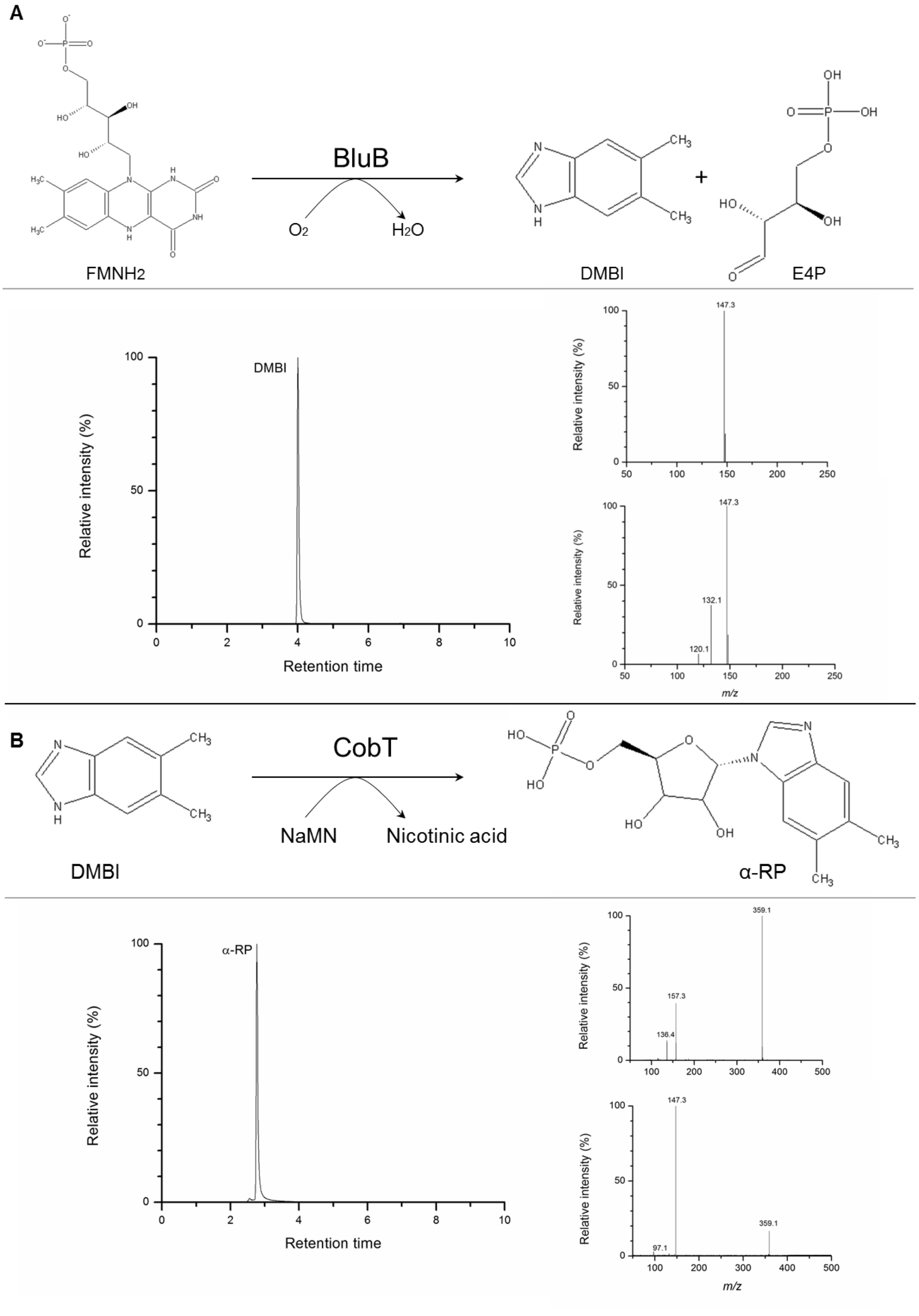

Figure S1 Reactions, retention times and fragmentation spectra of the BluB/CobT2 products: DMBI (A) and α-RP (B).

The reaction of BluB with reduced FMN and O_2 produced DMBI ($[M+H]^+$ *m/z* 147.3); reaction of CobT2 with DMBI and NaMN produced α-RP ($[M+H]^+$ *m/z* 359.1)

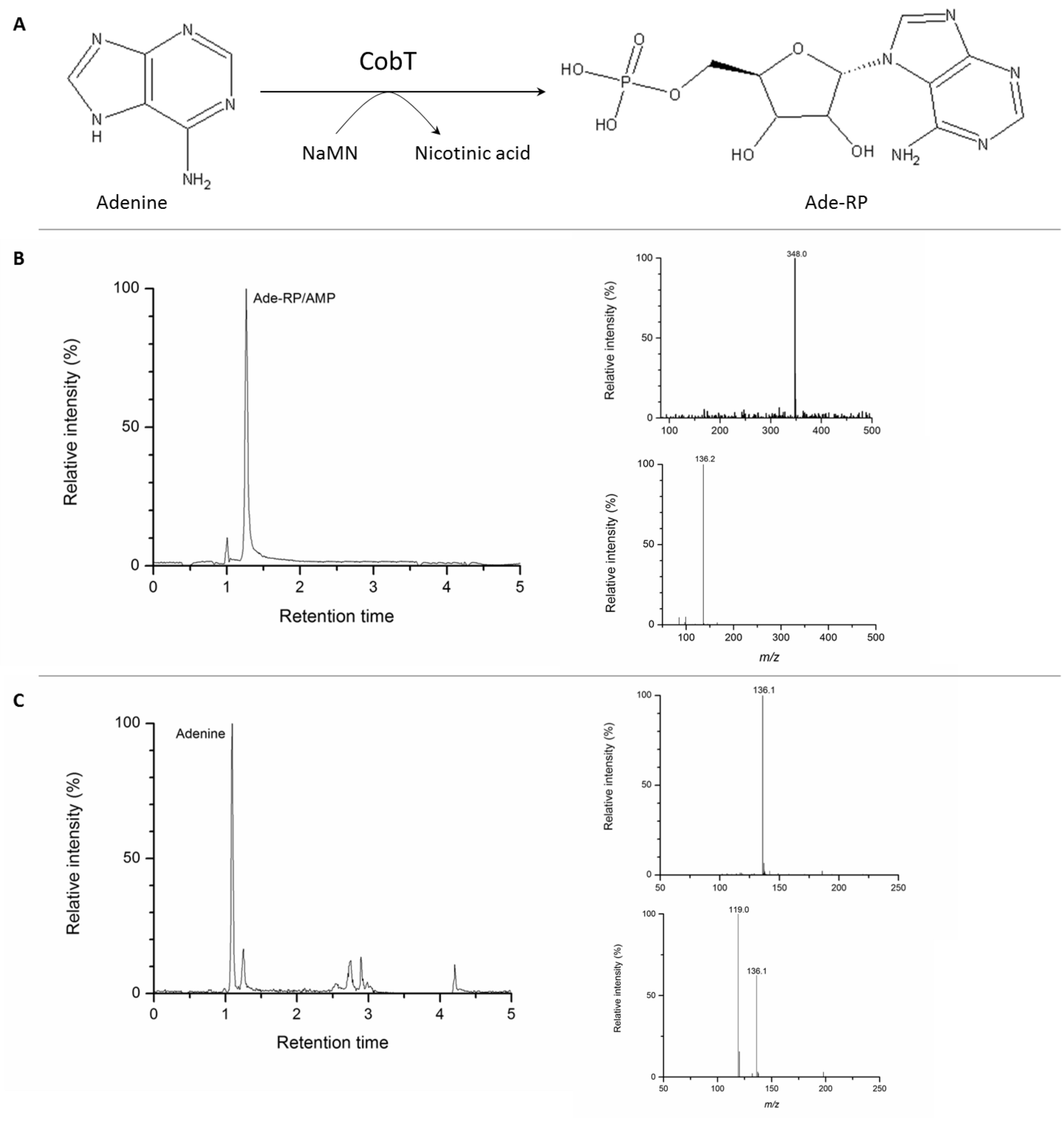

Figure S2 Reaction of CobT with adenine (A); retention time and fragmentation spectra of Ade-RP/AMP (B); retention time and fragmentation spectra of adenine (C).

Notably, Ade-RP ($[M+H]^+$ *m/z* 348.0) is indistinguishable from AMP, which is the compound found in our experiments based on the comparison with control reaction containing enzyme alone**.**

Comparison of BluB/CobT2 to other CobT proteins supports lack of activation of adenine

Studies on *S. enterica* CobT revealed that serine at position 80 interacts with the amino group (N10) and glutamine at position 88 interacts with the N3 ring nitrogen of adenine, stabilizing it in the active site [17; 39]. In CobT homologues, the amino acid corresponding to serine at position 80 in CobT of *Salmonella enterica* discriminates between adenine activation versus DMBI activation (Additional table 1). Small polar amino acids (serine and aspartic acid) interact with the N10 amino group of adenine and are found in those CobT enzymes that activate adenine. In contrast, large aromatic amino acids (phenylalanine, tyrosine, and tryptophan) would clash with the N10 amino group of adenine and are found in those CobT enzymes that activate DMBI but do not activate adenine. Additionally, the charged D80 excludes activation of the hydrophobic DMBI, which would otherwise leave an energetically unfavorable, unpaired buried charge. We propose the following rules to predict specificity: (i) a hydrophobic residue in position 80 implies activation of DMBI and exclusion of adenine, (ii) a polar residue in position 80 implies activation of adenine, (iii) a charged residue in position 80 implies exclusion of DMBI. Since changing glutamine to methionine at position 88 decreased the reaction with adenine and furthermore increased the reaction with DMBI in *S. enterica* CobT [17], we suggest that hydrophobic residue at this position has a supporting role in stabilization of DMBI, while uncharged polar residue does not exclude it.

Table S1 CobT homologues from various microorganisms and their activities. Amino acids in positions corresponding to S80 and Q88 of CobT from *Salmonella enterica* (SeCobT) are predictors of the activity.

Organism	Protein accession number	Activation of DMBI	Activation of adenine	Position corresponding to S80 of SeCobT	Position corresponding to Q88 of SeCobT	Reference
S. enterica	AAL20920.1	+	+	S	Q	[17]
L. reuteri	ABQ83934.1	+/- [a]	+	S	Q	[17]
S. meliloti 1021	CAC46463.1	+	-	F	M	[17]
P. denitrificans	WP_034795180.1	+	(-) [b]	F	M	[A1]
V. parvula DSM 2008 (Te3)	ACZ25278.1	+	+/-[a]	Y	Q	[17]
P. acidipropionici	AFV88734.1	-	+	D	M	Chamlagain et al., Unpubl.
P. freudenreichii subsp. *shermanii*	CBL56167.1	+	+/- [c]	F	V	This work
P. freudenreichii subsp. *freudenreichii*	AJQ90324.1	+ [c]	(-)	F	V	Chamlagain et al., Unpubl.
P. acnes SK137	YP_003581177.1	(+)	(-)	F	V	-
S. griseus	KIX33754.1	+	-	W	M	[A2]
K. setae KM-6054	WP_014134622.1	(+)	(-)	W	M	-

[a] Activates when heterologously expressed in *Si. meliloti*, no activation in parent organism *in vivo*

[b] Parentheses indicate activity predicted from CobT sequence

[c] Activation observed *in vivo* only

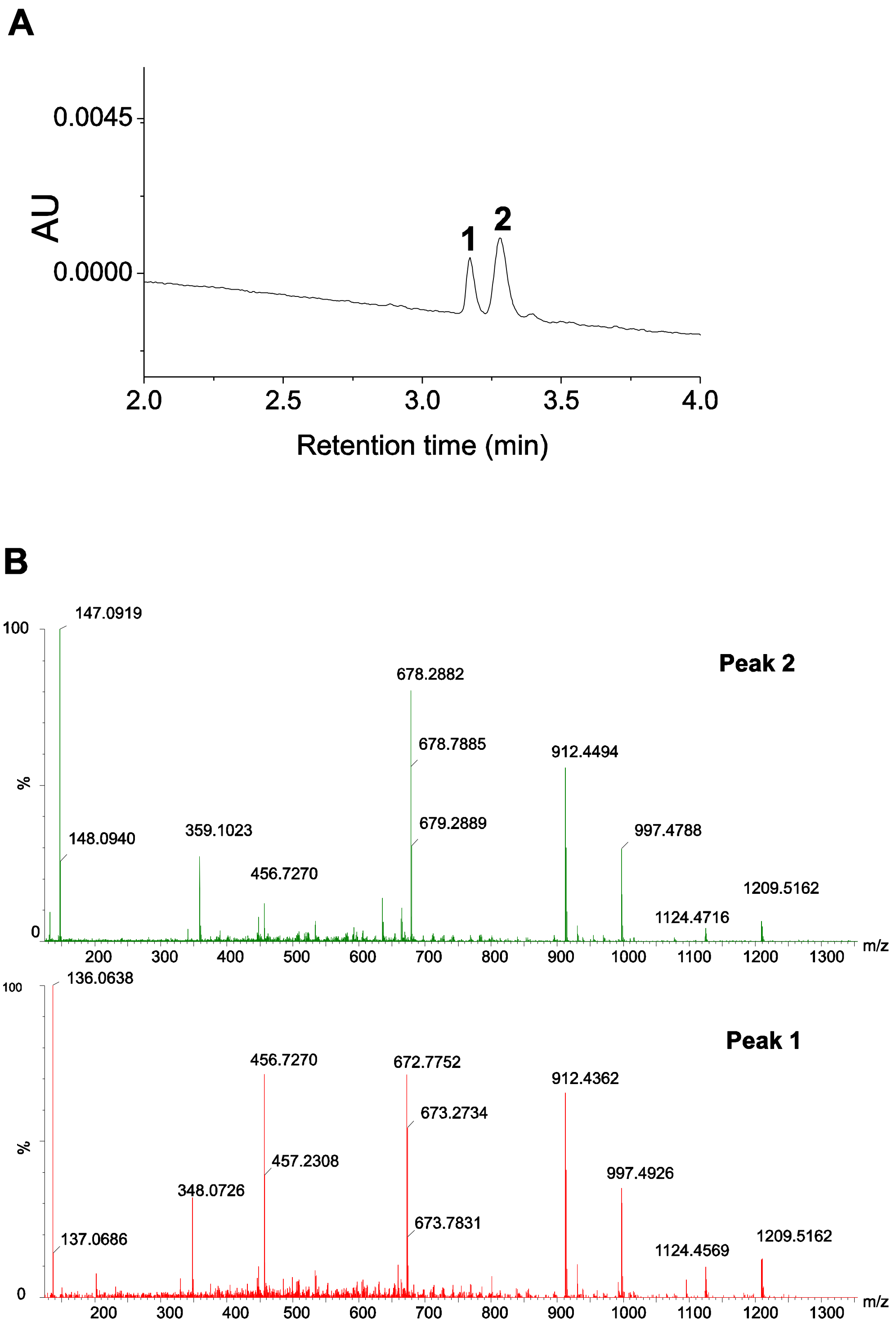

Figure S3 A) UHPLC-UV (361 nm) chromatogram of cobamide extract after purification on immunoaffinity column, confirmed with UHPLC-MS/MS to be pseudocobalamin (peak 1, retention time 3.17 min) and cyanocobalamin (peak 2, retention time 3.27 min).

B) The UHPLC-MS/MS spectra of peak 2 (cyanocobalamin) confirmed the presence of DMBI (*m/z* 147.0919) and α-RP (*m/z* 359.1023) and of peak 1 (pseudocobalamin) showed the presence of adenine (*m/z* 136.0638) and Ade-RP (*m/z* 348.0726). The parent doubly charged ions, *m/z* 678.2882 (cobalamin) and *m/z* 672.7752 (pseudocobalamin), were fragmented. The other fragments were identical.

The mobile phase contained 0.1% formic acid for the UHPLC-MS and the injection volume was 1 µL.

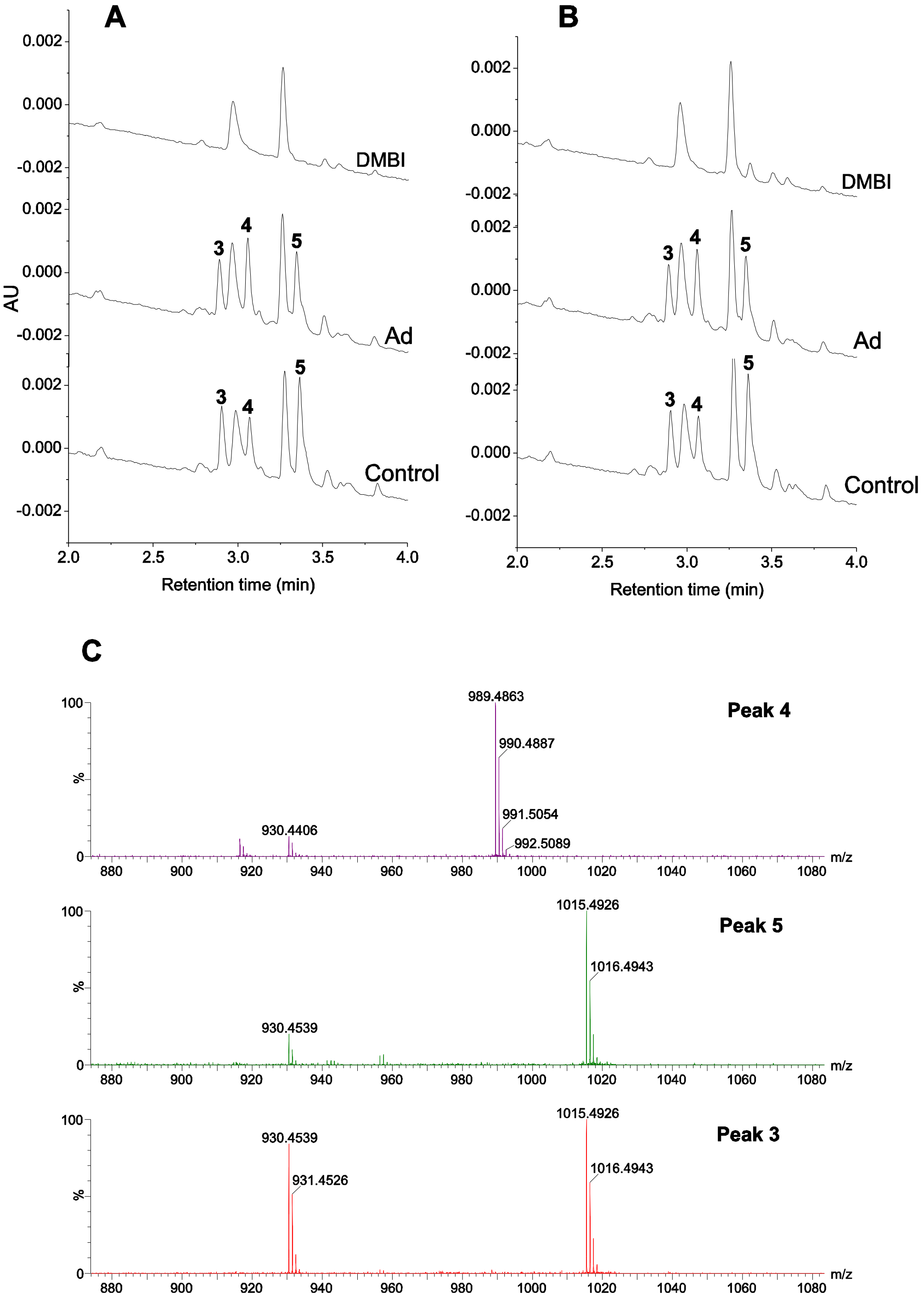

Figure S4 UHPLC-UV (361 nm) chromatograms of the flowthrough which passed through the immunoaffinity columns and the UHPLC-MS/MS spectra of cobinamides.

Extracts from *P. freudenreichii* cells grown under anaerobic (A) and microaerobic (B) conditions, with and without DMBI (100 µM) or adenine (100 µM). Three peaks (peak 3, peak 4 and peak 5) identified with UHPLC-MS/MS to be cobinamides (C) were seen only in cells of the cultures when no DMBI was added. The ions of the peak 3 and 5 had an *m/z* of 1015.4926 corresponding to $[M+H-(\alpha\text{-RP})]^+$ and of the peak 2 had an *m/z* of 989.4863 corresponding to $[M+H-(\alpha\text{-RP})\text{-CN}]^+$. Fragment ions with an *m/z* of 930.4406 corresponding to $[M+H-(\alpha\text{-RP})\text{-CN-Co}]^+$ were seen on MS/MS of the parent ions from each of the three peaks.

The mobile phase for UHPLC-UV and LC-MS contained 0.1% formic acid.

Table S2 Primers used for PCR amplification of the *bluB/cobT2* coding region: P1 and P2 with SgfI and PmeI restriction sites (marked with triangles); P3 and P4 used for screening of the *E. coli* KRX clones carrying pFN18A-bluB/cobT2 constructs.

P1	5’TTGCGAT ▼CGCGAGTGATGAGGCGCGTGATC3’
P2	5’TTGTTT▼AAACGCGCTGCGGGAGCGCTACT3’
P3	5’CGGATCCAGTGATGAGGCGCGTGATCCCGA3’
P4	5’ACGGAAGCTTGCGCTGCGGGAGCGCTACT3’

Additional references

A1 Cameron, B., Blanche, F., Rouyez, M. C., Bisch, D., Famechon, A., Couder, M., et al. Genetic analysis, nucleotide sequence, and products of two Pseudomonas denitrificans cob genes encoding nicotinate-nucleotide: dimethylbenzimidazole phosphoribosyltransferase and cobalamin (5'-phosphate) synthase. Journal of bacteriology, 1991, 173, 6066-6073.

A2 Perlman, D., & Barrett, J. M. Biosynthesis of cobalamins by cell suspensions of propionibacteria and streptomycetes. Journal of bacteriology, 1959, 78, 171.
